# Therapeutic Notes

**Published:** 1898-02-26

**Authors:** 


### THERAPEUTIC NOTES.

### Cystitis in Paraplegia.

Where in cases of paraplegia the bladder becomes foul, this condition must be remedied if the patient is to live; and there can be little doubt, says Dr. Vivian Poore, that the washing out of the bladder with solution of quinine, or boracic acid, or emulsion of iodoform (which is generally successful when the others fail) has added months or years to the life of many a chronic invalid.

